# Assessment of preoperative exercise capacity in hepatocellular carcinoma patients with chronic liver injury undergoing hepatectomy

**DOI:** 10.1186/1471-230X-13-119

**Published:** 2013-07-22

**Authors:** Masaki Kaibori, Morihiko Ishizaki, Kosuke Matsui, Richi Nakatake, Tatsuma Sakaguchi, Daiki Habu, Sawako Yoshiuchi, Yutaka Kimura, A Hon Kon

**Affiliations:** 1 Department of Surgery, Hirakata Hospital, Kansai Medical University, 573-191 Hirakata, Osaka, Japan; 2 Department of Nutritional Medicine, Graduate School of Human Life Science, Osaka City University, 545-8585 Osaka, Japan; 3 Department of Nutrition Management, Hirakata Hospital, Kansai Medical University, 573-191 Hirakata, Osaka, Japan; 4 Health Science Center, Hirakata Hospital, Kansai Medical University, 573-191 Hirakata, Osaka, Japan; 5 Masaki Kaibori, Department of Surgery, Hirakata Hospital, Kansai Medical University, 2-3-1 Shinmachi, 573-1191 Hirakata, Osaka, Japan

**Keywords:** Liver cancer, Chronic liver injury, Hepatectomy, Exercise capacity, BCAA/tyrosine ratio

## Abstract

**Background:**

Cardiopulmonary exercise testing measures oxygen uptake at increasing levels of work and predicts cardiopulmonary performance under conditions of stress, such as after abdominal surgery. Dynamic assessment of preoperative exercise capacity may be a useful predictor of postoperative prognosis. This study examined the relationship between preoperative exercise capacity and event-free survival in hepatocellular carcinoma (HCC) patients with chronic liver injury who underwent hepatectomy.

**Methods:**

Sixty-one HCC patients underwent preoperative cardiopulmonary exercise testing to determine their anaerobic threshold (AT). The AT was defined as the break point between carbon dioxide production and oxygen consumption per unit of time (VO_2_). Postoperative events including recurrence of HCC, death, liver failure, and complications of cirrhosis were recorded. Univariate and multivariate analyses were performed to evaluate associations between 35 clinical factors and outcomes, and identify independent prognostic indicators of event-free survival and maintenance of Child-Pugh class.

**Results:**

Multivariate analyses identified preoperative branched-chain amino acid/tyrosine ratio (BTR) <5, alanine aminotransferase level ≥42 IU/l, and AT VO_2_ <11.5 ml/min/kg as independent prognostic indicators of event-free survival. AT VO_2_ <11.5 ml/min/kg and BTR <5 were identified as independent prognostic indicators of maintenance of Child-Pugh class.

**Conclusions:**

This study identified preoperative exercise capacity as an independent prognostic indicator of event-free survival and maintenance of Child-Pugh class in HCC patients with chronic liver injury undergoing hepatectomy.

## Background

Major surgery has been shown to increase oxygen demand by about 40%, which may place severe stress on cardiopulmonary reserve [1]. Patients with high cardiopulmonary risk have traditionally been assessed using tests such as transthoracic echocardiography, dobutamine stress echocardiography, radionuclide ventriculography, and spirometry. However, these assessments have not been validated as preoperative screening tests, and provided mostly static measurements of cardiopulmonary performance [2-4]. Walking distance or ability to climb stairs have been used as subjective measurements of exercise tolerance, and have been shown to predict perioperative complications [5,6]. However, these measurements lack objectivity and do not detect silent cardiopulmonary abnormalities. Dynamic assessment of preoperative exercise capacity may be a useful predictor of short- and long-term postoperative prognosis. Cardiopulmonary exercise (CPX) testing measures oxygen uptake at increasing levels of work and predicts cardiopulmonary performance under conditions of stress, such as after surgery. In elderly patients undergoing major abdominal surgical procedures, the majority of deaths from cardiopulmonary complications occur in patients with an anaerobic threshold (AT) of <11 ml/min/kg [7,8].

Hepatocellular carcinoma (HCC) is the fifth most common cancer worldwide [9]. Maintenance of good perioperative nutrition and metabolism may improve the prognosis of patients with HCC undergoing hepatectomy [10,11]. To date, few studies have examined the usefulness of preoperative CPX testing in patients undergoing hepatectomy. In the present study, we aimed to clarify whether preoperative exercise capacity was related to event-free survival in HCC patients with chronic liver injury undergoing hepatectomy.

## Methods

### Patients

HCC patients with chronic hepatitis or cirrhosis who were scheduled for liver resection at Hirakata Hospital of Kansai Medical University (Osaka, Japan) between December 2008 and April 2010 were screened for inclusion this study. A total of 66 HCC patients underwent curative resection (defined as macroscopic removal of all tumor). There was no in-patient mortality. Sixty-one of the 66 patients were analyzed in this study, and the other 5 were excluded because they were followed up at other hospitals. All patients gave written informed consent for participation in this study. The study protocol was approved by the institutional ethics committee.

### Cardiopulmonary exercise testing

Patients underwent preoperative CPX testing using a bicycle ergometer with an incremental protocol (5.0, 7.5, and 10 W/min). Twelve-lead electrocardiography was used to monitor heart rate, ST segment deviation, and arrhythmias, at rest and continuously during the exercise and recovery periods. Blood pressure was recorded at rest and every 2 min during the exercise and recovery periods. Peak oxygen consumption per unit of time (VO_2_) was obtained from breath-by-breath analysis of expired air. Peak VO_2_ was defined as the highest mean value during exercise when the subject could no longer continue pedaling at 60 rpm. The AT, indicating the onset of metabolic acidosis, was defined as the break point between carbon dioxide production and VO_2_[12], or the point at which the ventilatory equivalent for oxygen and end-tidal oxygen partial pressure curves reached their respective nadirs before beginning to increase again [13]. Thus, AT was set at the time of maximum fat combustion [14]. The respiratory compensation point was set at the point at which the ventilatory equivalent for carbon dioxide was lowest before a systemic increase, and when the end-tidal carbon dioxide partial pressure reached a maximum and began to decrease [15]. Exercise was stopped when the patient requested it because of fatigue, pain, or headache, or if there was failure to maintain a speed greater than 40 rpm for more than 30 seconds despite encouragement.

### Clinical variables and surgery

Before surgery, each patient underwent conventional liver function testing and measurement of the indocyanine green retention rate at 15 min (ICGR15). Hepatitis virus infection screening was performed by testing for hepatitis B surface antigen (HBsAg) and hepatitis C virus antibody (HCVAb). Alpha-fetoprotein (AFP) and protein induced by vitamin K absence/antagonism-II (PIVKA-II) levels were measured in all patients. We used two methods to determine body composition: dual-energy X-ray absorptiometry (DEXA) [16] and bioelectrical impedance analysis (BIA) [17]. Total body mass, mineral-free lean mass (non-bone fat-free mass), fat mass, and truncal fat were measured by whole body DEXA. BIA was performed using the whole body 8-electrode approach with a 5-500 kHz multifrequency impedance analyzer (InBody720, BIOSPACE Co., Ltd, Tokyo, Japan). Intracellular body water (ICW), extracellular water (ECW), total body water (TBW), body cell mass, and ECW ratio (ECW/TBW) were measured.

Surgical procedures were classified according to the Brisbane terminology proposed by Strasberg et al. [18]. Anatomic resection was defined as resection of the tumor together with the related portal vein branches and corresponding hepatic territory, and was classified as hemihepatectomy (resection of half of the liver), extended hemihepatectomy (right trisectionectomy, or similar procedures on the left or for smaller resections), sectionectomy (resection of two Couinaud sub-segments [19]), or segmentectomy (resection of one Couinaud sub-segment). All other procedures were classified as non-anatomical resection, which was frequently performed for peripheral or central tumors. Peripheral tumors and those with extrahepatic growth were treated by partial hepatectomy because this procedure achieved a sufficient surgical margin. Central tumors located near the hepatic hilum or major vessels were treated by enucleation only, because it was too difficult and/or dangerous to remove enough liver tissue to obtain adequate margins. One consultant pathologist reviewed all specimens for histologic confirmation of the diagnosis. The width of the surgical margin was measured as the distance from the tumor edge to the resection line.

### Follow-up

Peri- and postoperative complications and deaths were recorded to determine morbidity and mortality following hepatectomy. Postoperative complications were defined and classified according to the modified Clavien system [20]. Briefly, Grade I was any deviation from the normal postoperative course that did not require special treatment, Grade II required pharmacological treatment, Grade III required surgical or radiological intervention without (IIIa) or with (IIIb) general anesthesia, Grade IV was any life-threatening complication involving dysfunction of one (IVa) or multiple (IVb) major organs, and Grade V was death. Postoperative liver-related events recorded included recurrence of HCC, HCC-related death, postoperative liver failure, and complications of cirrhosis requiring hospitalization (hepatic encephalopathy, uncontrollable pleural effusion or ascites, and rupture of esophageal or gastric varices). The Child-Pugh class of every patient was determined preoperatively and every 6 months postoperatively.

All surviving patients were followed up at least every 3 months after discharge. Follow-up included physical examination, liver function testing, chest radiographs to check for pulmonary metastases, and ultrasonography, computed tomography, or magnetic resonance imaging to check for intrahepatic recurrence. Chest computed tomography was performed if the chest radiograph showed any abnormalities. Bone metastases were diagnosed by bone scintigraphy.

When recurrence of HCC was detected by changes in tumor markers or on imaging, recurrence limited to the remnant liver was treated by transarterial chemoembolization, lipiodolization, re-resection, or percutaneous local ablative therapy such as radiofrequency ablation. When extrahepatic metastases were detected, active treatment was undertaken in patients with good hepatic functional reserve (Child-Pugh class A or B) and good performance status (0 or 1), while other patients were only given radiation therapy to relieve symptoms of bone metastases. Surgical resection was undertaken in patients with a solitary extrahepatic metastasis and no intrahepatic recurrence.

### Prognostic factors

We performed univariate and multivariate analyses of 35 clinical factors to identify independent variables related to postoperative event-free survival and postoperative maintenance of Child-Pugh class. The patient factors investigated were gender, age, body mass index, alcohol abuse, HBsAg, HCVAb, non-hepatitis B or C virus infection, diabetes mellitus, white blood count, lymphocyte count, insulin, homeostasis model assessment of insulin resistance, and liver function (including albumin, total bilirubin, aspartate aminotransferase [AST], alanine aminotransferase [ALT], prothrombin time, cholinesterase, platelet count, alkaline phosphatase, ICGR15, total cholesterol, low density lipoprotein cholesterol, transferrin, transthyretin, retinol-binding protein [RBP], transthyretin [TTR], branched chain amino acid [BCAA]/tyrosine ratio [BTR], and Child-Pugh class). The tumor factors investigated were AFP and PIVKA-II. The exercise parameters investigated were AT, VO_2_, and peak VO_2_. The body composition parameters investigated using DEXA were body mass, fat mass, fat-free mass, and whole-body mineral density. The body composition parameters investigated using BIA were ICW, ECW, TBW, protein, mineral, body fat mass, and body cell mass.

All the variables that were identified as significantly associated with event-free survival or maintenance of Child-Pugh class by univariate analyses were then examined using the Cox proportional hazards model to identify variables that were independently associated with event-free survival or maintenance of Child-Pugh class.

### Statistical analysis

Continuous variables are presented as mean ± standard deviation (SD). The significance of differences between groups was assessed using the chi-square test or Mann–Whitney *U-*test, as appropriate. The Kaplan-Meier method was used to calculate rates of event-free survival and maintenance of Child-Pugh class as of February 2012, and the significance of differences in survival rates was estimated using the generalized log-rank test. The Cox proportional hazards regression model (stepwise method) was used for multivariate analyses. In all analyses, *p* < 0.05 was considered statistically significant.

## Results

This study included 61 patients (45 male, 16 female; mean ± SD age = 70 ± 9 years). Table 1 shows the perioperative characteristics of HCC patients. Postoperative complications were observed in five patients: Grade II complications in three patients (ascites and/or pleural effusion) and Grade IIIa complications in two patients (intra-abdominal abscess). We followed these 61 patients until February 2012, with a median follow-up time of 24 months (range 12-36 months). We analyzed the prognostic factors associated with event-free survival and maintenance of Child-Pugh class. Postoperative events were defined as death due to recurrence of HCC, recurrence of HCC, intractable pleural effusion or ascites, gastrointestinal bleeding, or hepatic encephalopathy. Of the 61 patients, seven died from recurrence of HCC, 22 developed recurrence of HCC, two developed intractable pleural effusion or ascites, two developed gastrointestinal bleeding, and two developed hepatic encephalopathy.

**Table 1:** Perioperative characteristics of HCC patients

	
Age (years)	70 ± 9
Gender (male/female)	45/16
HBV/HCV/NBC	12/32/15
Child-Pugh class (A/B)	56/5
Diabetes mellitus (+/−)	8/53
WBC count (/μl)	5,000 ± 1,319
Lymphocyte count (/μl)	1,484 ± 580
ICGR15 (%)	16.1 ± 7.8
Albumin (g/dl)	3.8 ± 0.4
Total bilirubin (mg/dl)	0.81 ± 0.21
Cholinesterase (U/l)	232 ± 64
Triglyceride (mg/dl)	85 ± 43
Prothrombin time (%)	92 ± 12
Platelet count (×10^4^/μl)	15 ± 8
AST (U/l)	43 ± 27
ALT (U/l)	42 ± 28
RBP (mg/dl)	3.3 ±1.4
TTR (mg/dl)	16 ±6
BTR	5.09 ± 1.46
AFP (ng/ml)	994 ± 5,028
PIVKA-II (mAU/ml)	1,283 ± 2,209
Esophageal and/or gastric varices (+/−)	15/46
Surgical procedure (limited/anatomic)	35/26
Operation time (min)	329 ± 130
Operative blood loss (ml)	1,011 ± 1,351
Blood transfusion (+/−)	9/52
Tumor size (cm)	4.42 ± 4.09
Associated liver disease	
(normal/fibrosis or hepatitis/cirrhosis)	9/30/22
Morbidity (+/−)	5/56

Data represent the mean ± standard deviation or the number of patients. *HBV* hepatitis B virus, *HCV* hepatitis C virus, *NBC* non-hepatitis B or C virus, *WBC* white blood cell, *ICGR15* indocyanine green retention rate at 15 min; *ALT* alanine aminotransferase, *RBP* retinol binding protein, *TTR* transthyretin, *BTR* branched chain amino acid/tyrosine ratio, *AFP* α-fetoprotein, *PIVKA-II* protein induced by vitamin K absence/antagonism-II.

Child-Pugh class changed from class A preoperatively to class B postoperatively in nine patients, from class A to class C in one patient, and from class B to class C in one patient.

### Factors associated with event-free survival and maintenance of child-pugh class

The preoperative factors significantly associated with event-free survival on univariate analyses were preoperative ALT, albumin, RBP, BTR, platelet count, AT VO_2_, peak VO_2_, ICW, body cell mass, and total body protein (Table 2). The preoperative factors associated with maintenance of Child-Pugh class on univariate analyses were albumin, BTR, triglyceride, and AT VO_2_. Multivariate analyses (Cox proportional hazards model) of the factors associated with event-free survival on univariate analyses identified preoperative BTR <5, ALT ≥42 IU/l, and AT VO_2_ <11.5 ml/min/kg as independent prognostic indicators of event-free survival (Table 3). Multivariate analyses (Cox proportional hazards model) of factors associated with maintenance of Child-Pugh class on univariate analyses identified preoperative AT VO_2_ <11.5 ml/min/kg and BTR <5 as independent prognostic indicators of maintenance of Child-Pugh class.

**Table 2:** Results of univariate analyses of potential prognostic factors for event-free survival in HCC patients

**Variable**	**No. of patients**	**1-year survival rate (%)**	**3-year survival rate (%)**	***p *value**
Etiology				
HBV	13	91.7	52.1	0.1144
HCV	33	78.1	29.0	
NBC	15	86.7	54.2	
AST (IU/l)				
≤43	30	83.3	45.6	0.1961
>43	31	82.8	28.3	
ALT (IU/l)				
≤42	30	93.1	53.3	0.01
>42	31	73.3	22.2	
Albumin (g/dl)				
≥3.8	34	87.9	51.0	0.0226
<3.8	27	76.9	11.5	
RBP (mg/dl)				
≥3.3	32	86.7	46.9	0.0210
<3.3	29	77.8	77.8	
BTR				
≥5.0	33	87.5	53.0	0.0238
<5.0	28	77.8	16.2	
Platelet count (10^4^/μl)				
≥15	32	85.7	52.1	0.0355
<15	29	80.6	20.9	
AT VO_2_ (ml/min/kg)				
≥11.5	32	90.3	42.3	0.0266
<11.5	29	75.0	33.4	
Peak VO_2_ (ml/min/kg)				
≥16.5	32	87.1	50.3	0.0331
<16.5	29	78.6	10.8	
Intracellular body water (l/BW kg)				
≥0.33	32	90.3	35.8	0.0129
<0.33	29	75.0	30.3	
Body cell mass (kg/BW kg)				
≥0.47	32	90.3	34,0	0.0359
<0.47	29	75.0	33.3	
Total body protein (kg/BW kg)				
≥0.14	35	85.3	36.3	0.1071
<0.14	26	78.3	32.2	

*HBV* hepatitis B virus, *HCV* hepatitis C virus, *NBC* non-hepatitis B or C virus, *AST* aspartate aminotransferase, *ALT* alanine aminotransferase, *RBP* retinol binding protein, *BTR* branched chain amino acid/tyrosine ratio, *AT* anaerobic threshold, *VO*_*2*_ oxygen consumption, *BW* body weight.

**Table 3:** Results of multivariate analyses of potential prognostic factors for event-free survival in patients with HCC

**Variable**	**Coefficient**	**SE**	**Relative risk**	***p *value**
BTR (≥5 vs. < 5)	1.240	0.415	3.454	0.0028
ALT (≥42 vs. < 42 IU/l)	1.045	0.418	2.841	0.0124
AT VO_2_ (≥11.5 vs. < 11.5 ml/min/kg)	1.004	0.412	2.730	0.0148

*SE* standard error, *BTR* branched chain amino acid/tyrosine ratio, *ALT* alanine aminotransferase, *AT* anaerobic threshold, *VO*_*2*_ oxygen consumption.

**Figure 1:**
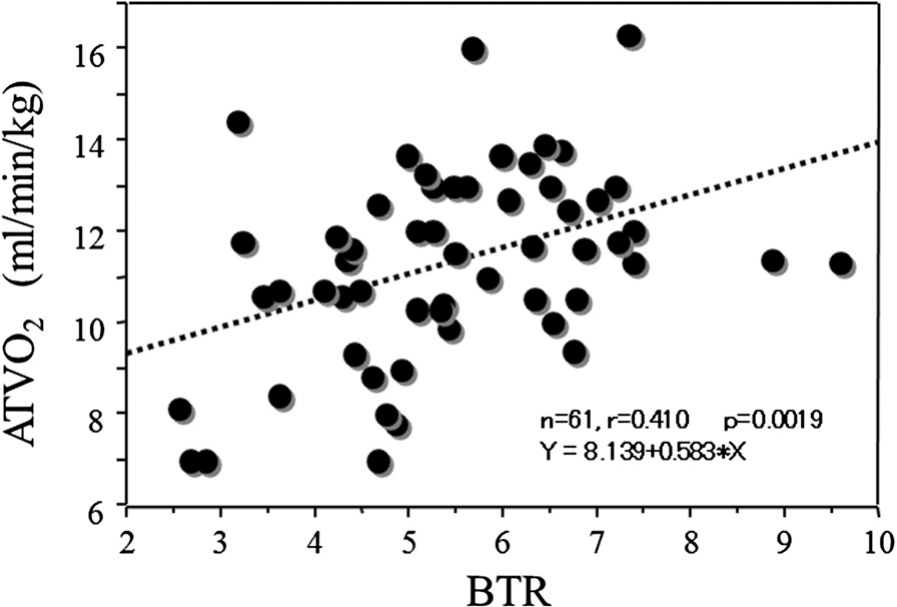
**Relationships between preoperative BTR and AT VO**_**2 **_**in HCC patients.** There was a significant correlation between BTR and AT VO_2_ (r = 0.410, Y = 8.139 + 0.583 * X, *p* = 0.0019). *BTR* branched chain amino acid/tyrosine ratio, *AT VO*_*2*_ anaerobic threshold oxygen consumption.

Figure 1 shows that there was a significant correlation between BTR and AT VO_2_ in HCC patients (r = 0.410, Y = 8.139 + 0.583 * X, *p* = 0.0019).

### Outcomes

**Figure 2:**
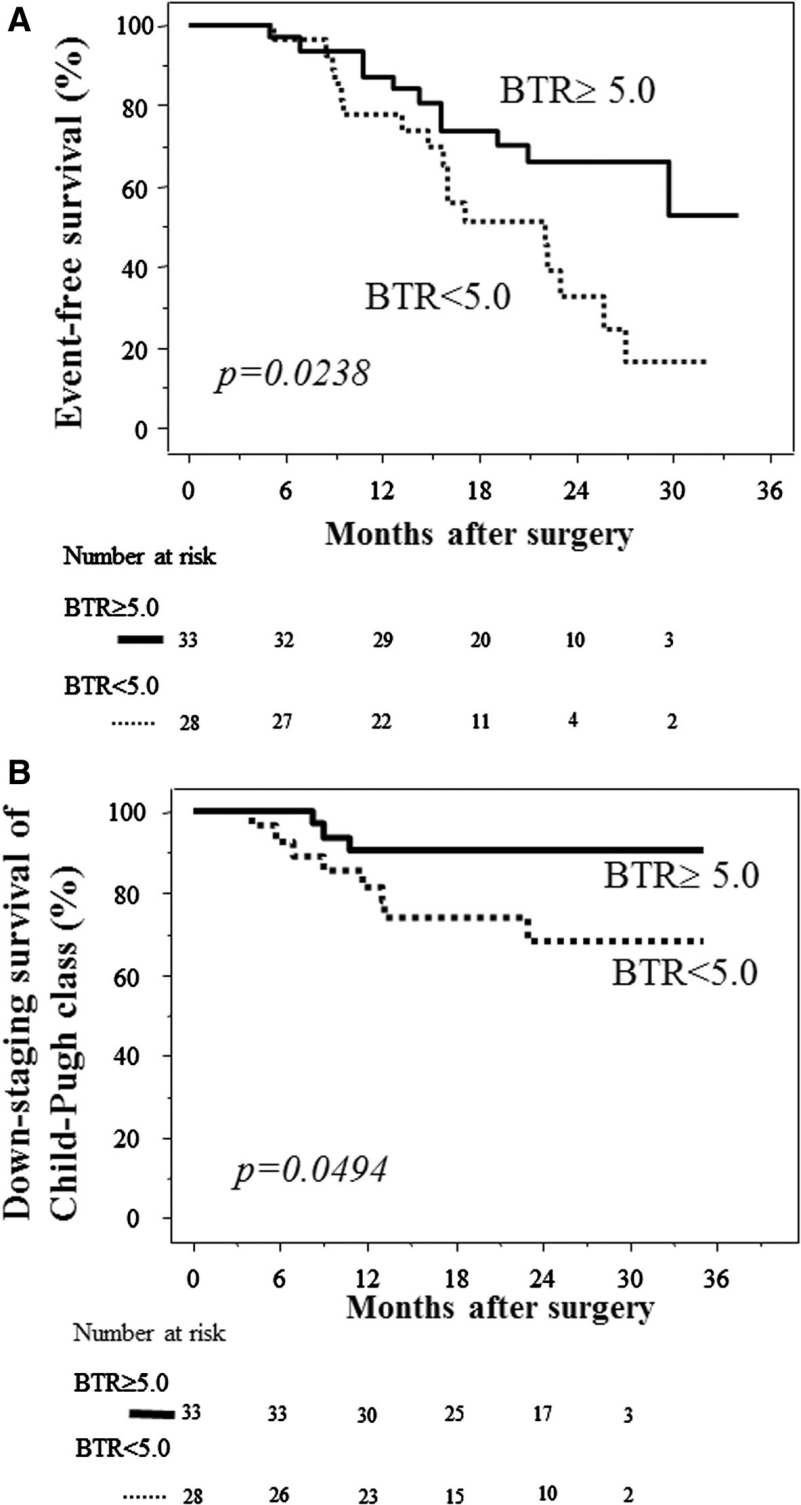
**Comparisons of event-free survival and maintenance of Child-Pugh class after hepatectomy between patients with preoperative BTR ≥5.0 and <5.0. (A)** Event-free survival. The survival rate was significantly higher in patients with preoperative BTR ≥5.0 (solid line) than BTR <5.0 (dotted line) (*p* = 0.0238). **(B)** Maintenance of Child-Pugh class. The rate of maintenance of Child-Pugh class was significantly higher in patients with preoperative BTR ≥5.0 (solid line) than BTR <5.0 (dotted line) (*p* = 0.0494). The numbers of patients at risk are shown below each graph. *BTR* branched chain amino acid/tyrosine ratio.

There was a significant difference in the event-free survival rate between patients with preoperative BTR ≥5.0 and <5.0 (*p* = 0.0238) (Figure 2A). There was also a significant difference in the rate of maintenance of Child-Pugh class between patients with preoperative BTR ≥5.0 and <5.0 (*p* = 0.0494) (Figure 2B).

**Figure 3:**
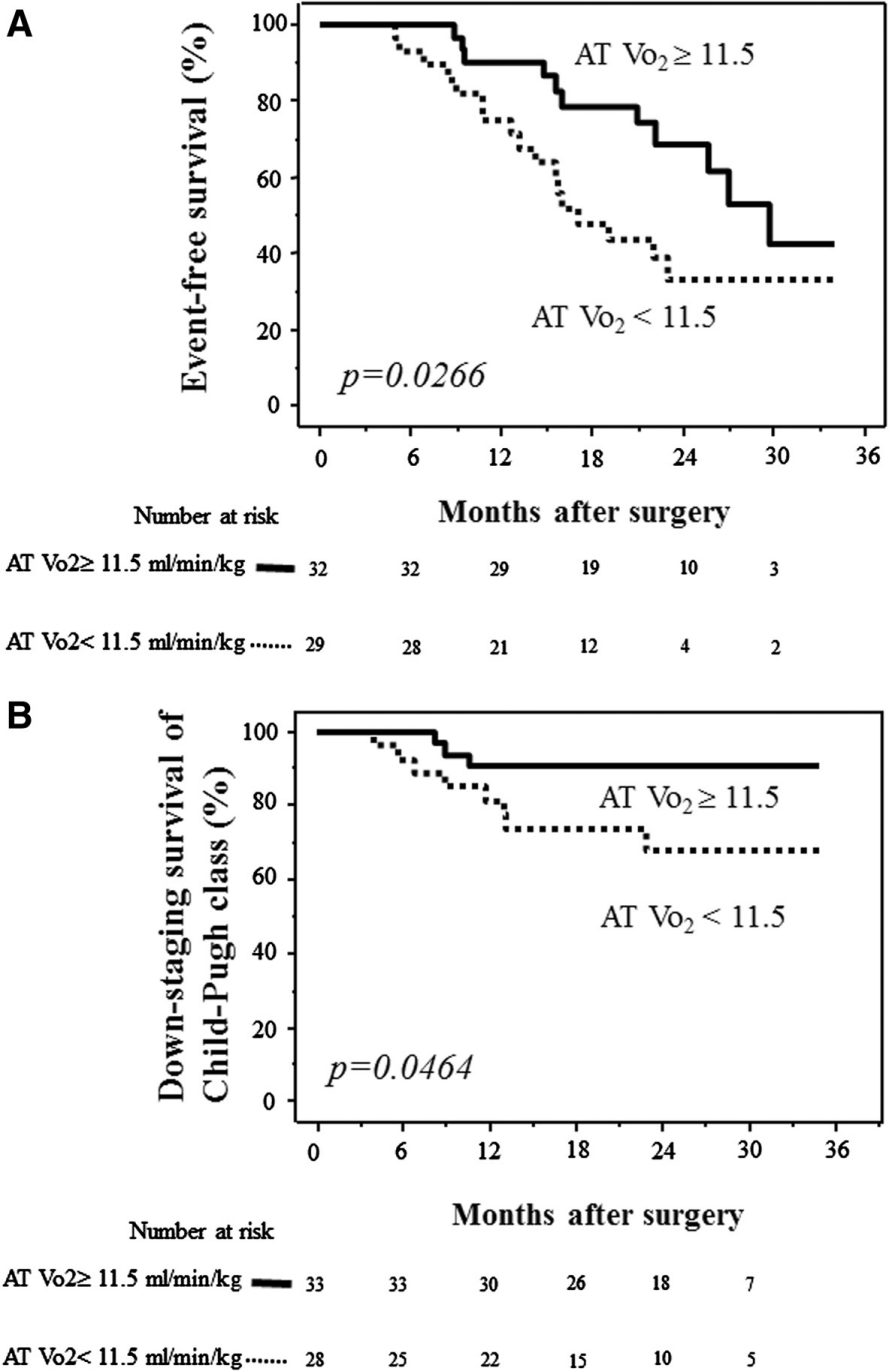
**Comparisons of event-free survival and maintenance of Child-Pugh class after hepatectomy between patients with preoperative AT VO**_**2 **_**≥11.5 ml/min/kg and <11.5 ml/min/kg. (A)** Event-free survival. The survival rate was significantly higher in patients with preoperative AT VO_2_ ≥11.5 ml/min/kg (solid line) than AT VO_2_ <11.5 ml/min/kg (dotted line) (*p* = 0.0266). **(B)** Maintenance of Child-Pugh class. The rate of maintenance of Child-Pugh class was significantly higher in patients with preoperative AT VO_2_ ≥11.5 ml/min/kg (solid line) than AT VO_2_ <11.5 ml/min/kg (dotted line) (*p* = 0.0464). The numbers of patients at risk are shown below each graph. *AT VO*_*2*_ anaerobic threshold oxygen consumption.

There was a significant difference in the event-free survival rate between patients with AT VO_2_ ≥11.5 and <11.5 ml/min/kg (*p* = 0.0266) (Figure 3A). There was also a significant difference in the rate of maintenance of Child-Pugh class between patients with AT VO_2_ ≥11.5 and <11.5 ml/min/kg (*p* = 0.0464) (Figure 3B).

## Discussion and conclusions

We examined the relationships among preoperative CPX parameters, postoperative events including recurrence of HCC, and change in Child-Pugh class in 61 patients undergoing hepatectomy. The variables derived from CPX testing included peak VO_2_, which is the maximum oxygen uptake at peak exercise. Previous studies indicated that peak VO_2_ was the most useful predictor of postoperative cardiopulmonary complications in patients undergoing radical esophagectomy with three-field lymphadenectomy [21] and patients undergoing surgical procedures for lung cancer [22-26]. The AT is defined as the point during exercise at which oxygen demand outstrips oxygen delivery and metabolism starts to become anaerobic. AT is a measure of the ability of the cardiopulmonary system to deliver adequate oxygen to tissues, and has the advantage of being independent of patient motivation. Reaching AT does not require high levels of physical stress and occurs well before peak VO_2_[27]. The usefulness of measuring AT has been assessed predominantly in elderly patients undergoing major surgical procedures, allowing the development of an operative risk grading and treatment protocol [7,8]. An AT cut-off of 11 ml/min/kg, which is internationally recognized, is currently used to select patients for enhanced recovery programs after colorectal surgery. However, AT has not been found to be useful in the assessment of cardiopulmonary fitness of patients undergoing esophagectomy only [21].

This study identified AT VO_2_ <11.5 ml/min/kg as an independent prognostic indicator of both event-free survival and maintenance of Child-Pugh class, indicating that CPX testing can be used to prospectively evaluate the cardiopulmonary function of HCC patients with chronic liver injury undergoing hepatectomy. It is possible that CPX testing can be used to predict postoperative recurrence of HCC or liver dysfunction. It has recently been reported that hepatic impairment, particularly cirrhosis, leads to secondary insulin resistance and hyperinsulinemia, resulting in the promotion of carcinogenesis. Management of insulin resistance is therefore critical in patients with chronic liver disease, to protect liver function and prevent hepatocarcinogenesis. Alterations in glucose metabolism also affect fat metabolism (production of lipid peroxide and reactive oxygen species) [28,29], which in turn may damage hepatocytes [30], leading to possible development of HCC [31]. It is difficult to manage insulin resistance associated with chronic liver disease, because restriction of caloric intake conflicts with the need to overcome malnutrition arising from hepatocellular damage. However, a reduction in body weight with exercise has been reported to be advantageous in obese patients with chronic liver disease [32]. The results of these studies and of our study indicate that hepatectomy for HCC patients with cirrhosis can be safely performed in patients with AT VO_2_ ≥11.5 ml/min/kg.

When liver function is impaired, metabolism of amino acids is also impaired. Consumption of BCAAs in skeletal muscle is increased to compensate for the lack of energy production by the liver [33], and aromatic amino acids (AAAs) are abundant. The balance of BCAAs and AAAs is known as the Fischer ratio [34]. The tyrosine level alone can be used instead of the AAA level to determine BTR [33]. BTR decreases in patients with liver dysfunction, such as chronic hepatitis or cirrhosis [35-37]. BTR can therefore be used as a marker of liver function in patients with liver disease [33,35-37]. BTR also has a high degree of correlation with other markers of liver function [36,37]. In the present study, we found that BTR was an independent prognostic indicator of recurrence of HCC or progressive liver dysfunction. Prolonged BCAA supplementation can therefore improve the prognosis of HCC patients who have undergone hepatectomy. It is important to pay attention to the preoperative BTR when planning perioperative care. We found a significant correlation between BTR and AT VO_2_ in HCC patients.

In conclusion, preoperative exercise capacity and BTR were identified as independent prognostic indicators of event-free survival and maintenance of Child-Pugh class in HCC patients with chronic liver injury undergoing hepatectomy. These results suggest that pre- and postoperative intervention with exercise therapy and BCAA supplementation may be beneficial in patients with chronic liver injury.

## Competing interests

The authors declare that they have no competing interests.

## Authors’ contributions

MK conducted the data analysis and drafted the manuscript. DH, YK and AHK conceived of the study, participated in its design, and helped draft the manuscript. MI, RN and TS participated in the study design and contributed to the data collection. KM participated in the study design and advised the analysis. SY contributed to study data collection. All authors contributed to the interpretation of the findings, and read and approved the final manuscript.

## Pre-publication history

The pre-publication history for this paper can be accessed here:

http://www.biomedcentral.com/1471-230X/13/119/prepub

## Synopsis for table of contents

In patients with hepatocellular carcinoma and hepatic dysfunction who underwent liver resection, preoperative exercise capacity and branched-chain amino acid/tyrosine ratio were identified as independent prognostic indicators of event-free survival and maintenance of Child-Pugh class. Improvement of preoperative exercise capacity may influence the short- and long-term postoperative prognosis in patients with chronic liver injury. Pre- and postoperative exercise therapy and branched-chain amino acid supplementation may be beneficial in these patients.

The study protocol was approved by the institutional ethics committee of Kansai Medical University (reference number: KMU H101036 and 080916).
